# Association of the bitter taste genes *TAS2R38* and *CA6* and breast cancer risk; a case-control study of Polish women in Poland and Polish immigrants in USA

**DOI:** 10.1371/journal.pone.0300061

**Published:** 2024-04-30

**Authors:** Dorota Łukasiewicz-Śmietańska, Dariusz Godlewski, Elżbieta Nowakowska, Andrzej Szpak, Elżbieta Chabros, Grzegorz Juszczyk, Jadwiga Charzewska, Dorothy Rybaczyk-Pathak

**Affiliations:** 1 Department of Nutrition and Nutritional Value of Food, National Institute of Public Health NIH- National Research Institute, Warsaw, Poland; 2 Center of Cancer Prevention and Epidemiology, OPEN, Poznań, Poland; 3 Wielkopolska Center of Oncology, Poznań, Poland; 4 Institute of Rural Health in Lublin, Lublin, Poland; 5 National Institute of Food and Nutrition, Warsaw, Poland; 6 National Institute of Public Health NIH- National Research Institute, Warsaw, Poland; 7 Department of Epidemiology and Biostatistics, Michigan State University, East Lansing, MI, United States of America; Center for Primary Care and Public Health: Unisante, SWITZERLAND

## Abstract

It is known that the perception of bitterness is mediated by type 2 bitter taste receptors (*TAS2Rs*). However, recent reports have suggested that the carbonic anhydrase 6 (*CA6*) gene may also influence bitterness sensing. Genetic variants in these genes could influence dietary intake of brassica vegetables, whose increased consumption has been observed in the literature, though inconsistently, to decrease breast cancer (BC) risk. We hypothesized that the estimated odds ratios (ORs) for the association between BC and taster diplotype (PAV/PAV) and/or genotype A/A, will be in the direction of increased BC risk, potentially due to reduced consumption of brassica vegetables. Using a case-control study of BC in Polish women in Poland (210 cases and 262 controls) and Polish immigrant women to USA (78 cases and 170 controls) we evaluated the association of the taster diplotypes in *TAS2R38* gene and genotypes in the *CA6* gene and BC risk in these two populations individually and jointly. No significant increase in risk was observed for the TAS2R38 PAV/PAV diplotype (tasters) in each population individually or in the joint population. For the *CA6* gene, in the joint population, we observed an increased BC risk for the combined G/A and G/G genotypes (non-tasters) vs A/A (tasters), OR = 1.41 (95% CI 1.04–1.90, p = 0.026) which after adjustment for False Discovery Rate (FDR), was not significant at p≤0.05 level. However, for the joint population and for the combined genotype of the two genes AVI/AVI+G* (non-tasters) vs. PAV/*+A/A (tasters), we observed a significant increase in BC risk, OR = 1.77 (95%CI 1.47–2.74, p = 0.01), for the non-tasters, which remained significant after FDR adjustment. In conclusion for the joint population and the joint effect for the two bitter sensing genes, we observed an increase in BC risk for the bitterness non-tasters, association which is in the opposite direction to our original hypothesis.

## Introduction

At the molecular level, the bitterness perception is mediated by type 2 bitter-taste receptors (TAS2Rs) [1–3]. Bitter taste receptors belong to a group of G protein-coupled receptors (GPCR). The most studied taste receptor is *TAS2R38 (Taste 2 receptor member 38)* which functions as a bitter taste receptor in the taste buds of human papillae. The 3 single nucleotide polymorphisms (SNPs) of *TAS2R38* (rs713598, rs1726866, rs10246939) result in 3 amino acid substitutions (A49P, A262V, V296I) and these 3 SNPs are in strong linkage disequilibrium consequently generating 2 common haplotypes: PAV—dominant taster variant, and AVI—recessive non-taster variant. However other combinations of these three SNPs are also possible, forming two uncommon haplotypes (frequency<5% AAV and AAI) and four rare haplotypes (frequency<1% PAI, PVI, AVV and PVV) [4–7]. PAV carriers are significantly more responsive than AVI carriers, to bitter taste of chemical compounds such as phenylthiocarbamide (PTC) and 6-n-propylthiouracil (PROP), used for identifying perception of bitter taste [8, 9]. The mentioned uncommon or rare haplotypes exhibit varying sensitivity to PTC and PROP which is positioned between the 2 common haplotypes [10, 11]. The research results show that the *TAS2R38* gene is responsible for about 50–85% of the variation in the PTC taste sensitivity trait, however, other genes can affect this trait as well [12].

One of the genes that can modify the bitter taste trait is the carbonic anhydrase VI gene *CA6*, which is responsible for the synthesis of a zinc metalloprotein -gustin- secreted by parotid, submandibular, and von Ebner glands [13–15]. Padiglia et al., observed that rs2274333 within *CA6* gene is responsible for an amino acid substitution at position Ser90Gly in the peptide which influences zinc binding to gustin and affects its enzymatic activity. This SNP has two variant alleles A and G. The A allele is associated with a more active isoform and G allele is connected with less functional isoform of the protein [16]. There are few studies on *CA6* gene function in the context of bitter taste, and findings are inconsistent. Calo et al. observed that the A/A genotype is more frequent in super tasters, while the G/G genotype is more frequent in non-tasters [16, 17]. Moreover, Melis et al. investigated the density of fungiform papillae on the anterior part of the tongue and they reported that rs2274333 has an influence on both fungiform papillae density and morphological changes such as size and shape [18]. The authors observed that the perception of PROP bitterness taste was statistically significantly higher in individuals with A/A genotype, however, A/G and G/G genotypes did not differ from each other in their PROP bitterness perception. These results were not confirmed by Feeney and colleagues who did not observe a relationship between rs2274333, the perception of PROP and the fungiform papillae density. They also did not observe the significantly higher frequency of the A/A genotype in tasters vs non-taster [19].

Brassica vegetables which abound in glucosinolates have a strong bitter taste [20]. Several studies reported that the individuals with PAV haplotype (*TAS2R38*) are more sensitive to the bitterness of these vegetables potentially leading to their lower consumption [21–26] although not all studies confirm this relationship [27, 28]. Additionally, it has been reported in the literature that reduced consumption of brassica vegetables is associated with increased risk of several cancers [29–35]. Therefore, the genetic variants of *TAS2R38*, by potentially modifying intake of these vegetables could impact cancer risk.

The distribution of these haplotypes has been evaluated in gastric, colorectal and intestinal cancers [6, 36–39]. To date no study has looked at the distribution of these haplotypes for breast cancer (BC). To address this gap in the literature, we will evaluate the distribution of these haplotypes and diplotypes among Polish women in the two regions in Poland: Poznan and Białystok and Polish immigrant women in USA.

The main purpose of our research was to assess if the frequency of PAV (tasters) and AVI (non-tasters) haplotypes in *TAS2R38* and, the frequency of the A and G allele in rs2274333 SNP in *CA6* gene are associated with BC status. We hypothesized that BC cases will have higher frequency of the PAV haplotype (tasters) and *CA6* A allele, which could impact BC risk, by reduced consumption of brassica vegetables, a mediator between these genotypes and BC. If the effect of bitter tasting genotypes on BC risk is mediated through the reduced consumption of cruciferous vegetables, then we would expect to observe a significant increase in BC risk for individuals with these genotypes [40].

## Materials and methods

### Subjects

The biological material analyzed in this publication comes from an American-Polish case-control study entitled "Breast Cancer in Women of Polish Ancestry" conducted (2000–2006) in Polish women in Poland and Polish immigrant women residing in USA, under the grant (No. R01 CA069670-NIH/NCI). Additional funding for collection of mouthwash rinse samples, from participant in the main study was obtained in 2004, under grants: "Breast Cancer; Gene Diet Interaction in Polish Women” (R03CA092838) and “Breast Cancer; Gene Diet Interaction—US Polish Migrants” (R03CA096436). Mouthwash rinse samples were collected between 2004–2006.

In Poland, breast cancer cases were identified by the Cancer Registries at: Greater Poland Cancer Centre (Wielkopolskie Centrum Onkologii in Poznań), the OPEN Cancer Prevention and Epidemiology Center in Poznań (Ośrodek Profilaktyki i Epidemiologii Nowotworów), and the Oncology Center in Białystok (Białostockie Centrum Onkologii). Polish immigrant breast cancer cases were identified by the Illinois State Cancer Registry, Springfield, IL and the SEER Cancer Registry at the Karmanos Cancer Institute, Detroit, MI.

Population based controls were randomly selected according to the age and place of residence of the BC cases for each participating Center, separately. In Poland, the selection of the sample was made using the National Population Registry (PESEL). In the USA, for the two regions, Cook County Illinois, and Detroit Metropolitan Area, Michigan, the control sample was selected by the Institute for Public Policy and Social Research (IPPSR), by random digit dialing, and supplemented by sample of women over the age of 65 identified through Health Care and Financing Administration (HCFA). Further details of the population based controls selection are described in publications [34, 41].

In the present study, mouthwash rinse samples were available for 789 women; 522 for Poland (235 cases and 287 controls) and 267 for USA (89 cases and 178 controls).

All women provided written consent for saliva samples collection and subsequent analysis of the samples. All protocols in this study were in accordance with the Declaration of Helsinki Principles, and all protocols were approved by Michigan State University (MSU-95-166D, MSU 02–632, MSU-02-631) and the Bioethics Commission at the National Institute of Food and Nutrition in Warsaw, Poland (4/175).

### DNA extraction and genotyping

DNA was extracted from the mouthwash using phenol/chloroform method described in the publication by Garcia-Closas [42]. The DNA was stored at -80°C in Poland and -20°C in USA, until use. Samples of DNA were diluted to 10 ng/μl, the PCR reaction was carried out in a volume of 20 μl. Four SNPs: three SNPs in *TAS2R38* (rs713598, rs1726866, rs10246939) and one SNP rs2274333 in *CA6* gene were determined using TaqMan® SNP Genotyping Assays (TaqPath™ ProAmp™ Master Mixes, ThermoFisher Scientific, MA, USA). The real-time polymerase chain reaction (RT‒PCR) was performed on StepOnePlus instrument (Applied Biosystems) using the run method recommended by the manufacturer. For quality control, 10% of duplicate DNA samples were analysed with the same process.

For the purposes of the analyses of this article the A/A genotype will be referred to as "tasters" while genotypes with at least one G allele will be referred to as "non-tasters". There is no such designation in the literature regarding the *CA6* gene. However, Padiglia et al. suggested that the G allele is less functional and more prevalent among individuals categorized as non-tasters in terms of responsiveness to the chemical compound PROP. In that study, the authors consider individuals with the A/G genotype as "medium tasters" meaning they have a moderate PROP responsiveness compared to those with the A/A genotype [16]. Since our hypothesis is strongly related to the perception of bitter taste, we decided to classify individuals with the intermediate genotype (A/G) as non-tasters to ensure that the tasters group consists of individuals who strongly perceive bitterness. Furthermore, another argument supporting this decision is that Melis et al. indicated that bitterness perception to PROP was significantly higher in individuals with genotype A/A than in those with the other genotypes, but not different between G/G and A/G [18].

### Statistical analysis

The observed genotype frequencies of all SNPs were tested for deviation from Hardy–Weinberg equilibrium (HWE) in controls in Polish and Polish immigrant populations separately by the χ^2^test. Haplotypes in *TAS2R38* were estimated using the PHASE computer program PHASE Microsoft Windows (version 2.1.1) [43, 44].

We viewed the two groups of women, as independent samples from the same target population, and thus replicas of each other. Therefore, the differences in the distribution between cases and controls, of the haplotypes in the *TAS2R38* gene, and allele in the *CA6* gene, were evaluated using χ^2^test for independence of attributes, first separately for women in Poland and Polish immigrant women, and then jointly for both groups. The homogeneity of the haplotypic ORs and allelic ORs for the two populations were tested by the Breslow-Day test for homogeneity of ORs. The association between the genotypes and breast cancer risk was estimated using an unconditional logistic regression to compute the odds ratios (OR), 95% confidence intervals (95% CI) and p-values, separately for each group of women and then for both groups jointly [45]. Where appropriate, we applied the False Discovery Rate (FDR) adjustment for multiple comparisons [46, 47]. A p-value ≤0.05 was considered statistically significant. All statistical analyses were performed using SAS version 9.4 (SAS Inc. Cary, NC, USA).

## Results

We genotyped all 789 participants in this study. However, 15 samples (7 in Poland and 8 in USA) were excluded due to missing identification of one of the four tested SNPs. Therefore, our haplotype analyses are based on 774 participants, 515 participants in Poland (232 cases and 283 controls), and 259 in USA (83 cases and 176 controls).

All genetic variants in *TAS2R38* (rs713598, rs1726866, rs10246939) and *CA6* (rs2274333), were in Hardy-Weinberg equilibrium (p>0.05, data not shown), in controls both in Poland and USA.

### Frequency of haplotypes in *TAS2R38* gene and alleles in *CA6* gene

The most frequently observed haplotypes in *TAS2R38* gene were PAV and AVI both in Polish women in Poland and Polish immigrant women in USA. We also observed uncommon and rare haplotypes, such as AVV, AAI and AAV. However, we excluded subjects with at least one these haplotypes from our analyses, because: 1) it has been observed in the literature [10, 11], that their perception of bitterness is intermediate between the common haplotypes, and 2) when we combined them in a way described by Carrai (dominance assumption of the taster haplotype) [6], our results did not change in terms of level of association with BC and statistical significance of the odds ratios. Distribution of all haplotypes for *TAS2R38*, including the uncommon and rare ones, is provided in S1 Table. After exclusions, our sample size for Poland was 472 (210 cases and 262 controls), and USA 248 (78 cases and 170 controls), for a total number of 720 participants. Since only women with common haplotypes (PAV and AVI) were included in our subsequent analyses of *TAS2R38* gene, and we planned to assess the joint effect of the two genes, for *CA6* genotype calculations, only individuals with PAV and AVI haplotypes were included. The observed frequencies of haplotypes in *TAS2R38* gene without the uncommon or rare haplotypes and the distribution of the allele for *CA6* gene are presented separately for Polish women in Poland, Polish immigrant women to USA and jointly for the two groups in Table 1.

**Table 1:** Frequency of haplotypes in *TAS2R38* gene (without the rare haplotypes) and alleles in *CA6* gene.

Haplotype	Polish women in Poland		Polish Immigrant women in USA		Combined groups Poland and USA	
TAS2R38 (%)	Cases n = 210	Controls n = 262	Cases n = 78	Controls n = 170	Cases n = 288	Controls n = 432
PAV	0.381	0.408	0.423	0.462	0.392	0.429
AVI	0.619	0.592	0.577	0.538	0.608	0.571
χ^2^ (1df)	0.73, p = 0.39		0.65, p = 0.42		1.95, p = 0.16	
Allelle CA6 (%)	Cases n = 210	Controls n = 262	Cases n = 78	Controls n = 170	Cases n = 288	Controls n = 432
A	0.662	0.706	0.641	0.726	0.656	0.714
G	0.338	0.294	0.359	0.274	0.344	0.286
χ^2^ (1df)	2.12, p = 0.15		3.72, p = 0.054		5.42, **p = 0.02**	

Unadjusted p-values from chi-square tests

We observed that for the two groups of women, as well as for the combined group, the distribution of the PAV and AVI haplotypes did not differ between cases and controls. For the *CA6* allele, we observed that cases, consistently in the two groups of women had a lower frequency of A allele relative to controls. This difference was not statistically significant at a p≤0.05 level, for women in Poland (χ^2^_1_ = 2.12, p = 0.15) as well as for the immigrant group (χ^2^_1_ = 3.72, p = 0.054). However, for the combined group the difference in the distribution of the alleles in *CA6* gene differed significantly between cases and controls (χ^2^_1 =_ 5.42, p = 0.02), and remained significant after adjustment for FDR, assuming independence of null hypothesis testing for the two Polish population.

### Associations of the *TAS2R38* and *CA6* with breast cancer risk

To examine the relationship between risk of breast cancer and *TAS2R38* and *CA6* gene individually and for the joint genotype we ran logistic regression to obtain odds ratios for breast cancer status for individual diplotypes of the *TAS2R38* and individual genotypes of the *CA6* gene. For evaluation of the joint effect of the two genes, we chose to combine diplotypes with at least one PAV haplotype, which is a dominant (taster) haplotype with PAV/PAV diplotype participants. For the *CA6* gene the two genotypes of G/A and G/G were combined, since we did not observe significant differences in their association with BC when tested individually, and the observation by Melis [18], that their bitterness perception to PROP did not differ. We performed all analyses for Polish women in Poland, Polish immigrant women to USA and jointly for the two groups (Tables 2–4).

**Table 2:** Associations of the *TAS2R38* and *CA6* with breast cancer in Polish women in Poland.

TAS2R38	Total n = 472	Cases n = 210	Control n = 262	OR crude	(95% CI)	p-value
PAV/PAV	73	30	43	1	1	
PAV/AVI	228	100	128	1.12	(0.66–1.91)	0.678
AVI/AVI	171	80	91	1.26	(0.72–2.19)	0.414
PAV/* vs. AVI/AVI	472	130 vs 80	171 vs 91	1.16	(0.79–1.67)	0.450
CA6 rs2274333	Total	Cases	Control	OR crude	(95% CI)	p-value
A/A	220	91	129	1	1	
G/A	208	96	112	1.22	(0.83–1.78)	0.318
G/G	44	23	21	1.55	(0.81–2.97)	0.184
A/A vs. G*	472	91 vs 119	129 vs 133	1.27	(0.88–1.83)	0.202
Combined genotype (TAS2R38+CA6)	Total	Cases	Control	OR crude	(95% CI)	p-value
PAV/*+A/A	137	60	77	1	1	
PAV/*+G*	164	70	94	0.96	(0.61–1.51)	0.846
AVI/AVI+A/A	83	31	52	0.77	(0.44–1.34)	0.347
AVI/AVI+G*	88	49	39	1.61	(0.94–2.77)	0.083

n:number of subjects, OR:Odds ratio, CI: Confidence interval, p-value: unadjusted for multiple comparisons—none were significant at 0.05 level.

PAV/*: PAV/PAV+PAV/AVI; G*: G/A+G/G; PAV/*+A/A: [TAS2R38 (PAV/PAV+PAV/AVI)+CA6 (A/A)]

PAV/*+G*: [TAS2R38 (PAV/PAV+PAV/AVI)+CA6 (G/A+G/G)]; AVI/AVI+A/A: [TAS2R38 (AVI/AVI)+CA6 (A/A)]; AVI/AVI+G*: [TAS2R38 (AVI/AVI) + CA6 (G/A+G/G)]

**Table 3:** Associations of the *TAS2R38* and *CA6* with breast cancer in Polish immigrant women in USA.

TAS2R38	Total n = 248	Cases n = 78	Control n = 170	OR crude	(95% CI)	p-value
PAV/PAV	51	14	37	1	1	
PAV/AVI	121	38	83	1.21	0.59–2.50	0.606
AVI/AVI	76	26	50	1.37	0.63–2.99	0.422
PAV/* vs. AVI/AVI	248	52 vs 26	120 vs 50	1.20	0.68–2.13	0.534
CA6 rs2274333	Total	Cases	Control	OR crude	(95% CI)	p-value
A/A	124	32	92	1	1	
G/A	99	36	63	1.64	0.93–2.92	0.090
G/G	25	10	15	1.92	0.78–4.69	0.155
A/A vs. G*	248	32 vs 46	92 vs 78	1.70	0.99–2.92	0.057
Combined genotype (TAS2R38+CA6)	Total	Cases	Control	OR crude	(95% CI)	p-value
PAV/*+A/A	91	23	68	1	1	
PAV/*+G*	81	29	52	1.65	0.86–3.18	0.135
AVI/AVI+A/A	33	9	24	1.11	0.45–2.73	0.822
AVI/AVI+G*	43	17	26	1.93	0.89–4.19	0.095

n:number of subjects, OR:Odds ratio, CI: Confidence interval, p-value: unadjusted for multiple comparisons—none were significant at 0.05 level.

PAV/*: PAV/PAV+PAV/AVI; G*: G/A+G/G; PAV/*+A/A: [TAS2R38 (PAV/PAV+PAV/AVI)+CA6 (A/A)]

PAV/*+G*: [TAS2R38 (PAV/PAV+PAV/AVI)+CA6 (G/A+G/G)]; AVI/AVI+AA: [TAS2R38 (AVI/AVI)+CA6 (A/A)]

AVI/AVI+G*: [TAS2R38 (AVI/AVI) + CA6 (G/A+G/G)]

**Table 4:** Associations of the *TAS2R38* and *CA6* with breast cancer in combined groups: Polish women in Poland and Polish immigrant women in USA.

TAS2R38	Total n = 720	Cases n = 288	Control n = 432	OR crude	(95% CI)	p-value
PAV/PAV	124	44	80	1	1	
PAV/AVI	349	138	211	1.19	(0.78–1.82)	0.43
AVI/AVI	247	106	141	1.37	(0.88–2.14)	0.17
PAV/* vs. AVI/AVI	720	182 vs 106	291 vs 141	1.2	(0.88–1.64)	0.25
CA6 rs2274333	Total	Cases	Control	OR crude	(95% CI)	p-value
A/A	344	123	221	1	1	
G/A	307	132	175	1.36	(0.99–1.86)	0.06
G/G	69	33	36	1.65	(0.98–2.77)	0.06
A/A vs. G*	720	123 vs 165	221 vs 211	1.41	(1.04–1.90)	**0.026**
Combined genotype (TAS2R38+CA6)	Total	Cases	Control	OR crude	(95% CI)	p-value
PAV/*+A/A	228	83	145	1	1	
PAV/*+G*	245	99	146	1.18	(0.82–1.72)	0.89
AVI/AVI+A/A	116	40	76	0.92	(0.58–1.47)	0.73
AVI/AVI+G*	131	66	65	1.77	(1.47–2.74)	**0.010**

n:number of subjects, OR:Odds ratio, CI: Confidence interval, p-value: unadjusted for multiple comparisons, except for the combined taster genotype vs. combined non-taster genotype, which meets statistical significance of 0.0167, when FDR adjusted.

PAV/*: PAV/PAV+PAV/AVI; G*: G/A+G/G; PAV/*+A/A: [TAS2R38 (PAV/PAV+PAV/AVI)+CA6 (A/A)]

PAV/*+G*: [TAS2R38 (PAV/PAV+PAV/AVI)+CA6 (G/A+G/G)]; AVI/AVI+AA: [TAS2R38 (AVI/AVI)+CA6 (A/A)]

AVI/AVI+G*: [TAS2R38 (AVI/AVI) + CA6 (G/A+G/G)]

### For Polish women in Poland

For Polish women in Poland, the odds ratios (ORs) for *TAS2R38* diplotype and *CA6* genotype in SNP rs2274333 and for their joint distribution, are presented in Table 2. No significant association was observed between breast cancer status and *TAS2R38* diplotypes or for *CA6* gene. When testing the joint effect of both genes on breast cancer status, our reference category was defined as PAV/*+A/A (tasters) vs. all other joint combinations of the two genes as shown. We observed that although carriers of AVI/AVI G* (the non-tasters on both individual genes) relative to tasters showed an increase in breast cancer risk, the observed OR did not reach statistical significance at p≤0.05 level (OR = 1.61, 95% CI 0.94–2.77; p = 0.083).

### Polish immigrant women in USA

For immigrant women in USA, the results are similar to those observed in Poland, with no statistically significant associations, at p≤0.05 level for BC risk and *TAS2R38* or *CA6* gene individually (Table 3). For the two genes combined, again we observed similar pattern to that in Poland. Individuals with AVI/AVI+G* genotype vs. tasters, had an increase in breast cancer risk, which was not statistically significant at p≤0.05 level (OR = 1.93; 95% CI 0.89–4.19; p = 0.095).

### Combined population of Polish women–Poland and US immigrant women

Given the that we considered the two groups of women, as independent samples from the same target population, and the similar patterns of associations, for women in Poland and Polish immigrant women in USA, and the Breslow-Day test for homogeneity of ORs for the distribution of haplotypes and alleles as presented in Table 1, was not significant, we combined the two groups of women, to increase our sample size. The results of our analysis for the combined group, are presented in Table 4. For the *TAS2R38* gene no significant associations with BC status were observed. However, for the *CA6* gene, when individual genotypes were compared, the comparison of the G/G genotype (homozygous for non-tasters) to the reference A/A genotype, showed an increase in risk, which was not significant at p≤0.05 level (OR = 1.65; 95% CI 0.98–2.77; p = 0.06). When the homozygous non-tasters, were combined with the heterozygous non-tasters, forming G* genotype, and compared to A/A (tasters), we observed a statistically significant increase in breast cancer risk (OR = 1.41; 95% CI 1.04–1.90; p = 0.026). However, when adjusted for FDR, the unadjusted observed p-value did not maintain statistical significance.

For the combined genes *TAS2R38* and *CA6*, the genotype AVI/AVI+G* (non-tasters), relative to the reference group of PAV/*+A/A (tasters) showed a statistically significant association with breast cancer status (OR = 1.77;95% CI 1.47–2.74; p = 0.010). Significance was maintained after adjustment for FDR.

## Discussion

In our study, we hypothesized that women with the genotype sensitive to bitter taste, PAV/PAV or A/A, will be sensitive to the bitterness present in the Brassica vegetables and potentially will consume less cruciferous vegetables. Consequently, they will not benefit from the anticarcinogenic properties of these vegetables, thus increasing their risk of developing breast cancer. If the association between lower consumption of cruciferous vegetables and increased cancer risk was mediated by their ability to taste bitterness, then we potentially could observe an association of the bitter tasting genotypes with increased cancer risk.

According to our hypothesis we expected to observe a higher frequency among cases of bitterness tasters, PAV haplotype in the *TAS2R38* gene and A allele in the *CA6* gene. However, our results showed that the distribution of PAV haplotype did not differ between cases and controls. For the A allele (taster) in *CA6* gene we observed a result in the opposite direction than expected. In the two groups of women individually, we observed lower frequency of A allele in cases relative to controls, although this difference did not reach statistical significance neither in Poland (66.2% vs. 70.6%, χ^2^1 = 2.12, p = 0.15) nor in the immigrant group (64.1% vs. 72.6%, χ^2^1 = 3.72, p = 0.054). For the two groups of women combined, thus larger sample size, we did observe a significantly lower frequency of A allele for the cases (65.6% vs. 71.4%, χ^2^_1 =_ 5.42, p = 0.02).

When evaluating associations (ORs) between the diplotypes of the *TAS2R38* gene and breast cancer risk, no significant associations were observed in Polish women in Poland, immigrant women to US and the combined group of women. For the *CA6* gene, when evaluating the genotype, our results again showed associations with breast cancer risk which were in opposite direction from what we initially hypothesized. Individuals with genotype of at least one G allele (non-taster), relative to A/A (tasters), showed an increase in risk of BC risk, which did not reach statistical significance at a p≤0.05 level. After combining the two groups of women (Poland and immigrants to US), the odds ratio for G* vs A/A (non-tasters vs. tasters) did reach unadjusted statistical significance, (OR = 1.41; 95% CI 1.04–1.90, p = 0.026), which when FDR adjusted was no longer statistically significant at p≤0.05 level. However, for the combined group of women, and for the combined genotype of AVI/AVIG*vs PAV*A/A (non-tasters vs. tasters), we observed an increase in risk of breast cancer (for the non-tasters), which was statistically significant (OR = 1.77; 95% CI 1.47–2.74 p = 0.01), and remained significant after FDR adjustment. However, due to the relatively small sample size of the study, and that the studied genes have a small effect on BC risk, we did not have sufficient power to detect several of our observed small effects, with our current sample size as statistically significant at p≤0.05.

Literature on the relationship between cancer risk and *TAS2R38* gene is inconsistent. In our study, we did not observe any statistically significant associations between diplotypes in TAS2R38 gene and breast cancer risk. Our observed direction and magnitude of the OR, was however, like that observed in the study by Carrai for colorectal (CRC) cancer [6]. Their study evaluated the association between the risk of colorectal (CRC) cancer and diplotypes in the TAS2R38 gene in German and Czech populations. They observed an increased risk of CRC with AVI/AVI diplotype (non-tasters), relative to PAV/PAV (tasters), which did reach statistical significance, in the combined populations (OR = 1.33; 95% CI 1.03–1.77 p = 0.027) [6]. In our study, for our combined population, we also observed that the OR for the AVI/AVI vs PAV/PAV comparison (non-tasters vs. tasters) was of similar magnitude (OR = 1.37; 95% CI 0.88–2.14, p = 0.17), however, it did not reach statistical significance at p≤0.05 level. The sample size in Carrai study was much larger; it included 2535 subjects (1203 cases 1332 controls).

Another study in Japanese population of gastrointestinal cancer and *TAS2R38* gene also reported a significant positive association between gastrointestinal cancer risk and the AVI/AVI diplotype (the non-tasters) vs. PAV/PAV, with OR = 2.04 (95% CI 1.095–3.815 p = 0.024) [39].

A null association, similar to ours, was observed for *TAS2R38* gene diplotypes and colorectal adenoma in a multiethnic population of Japanese American, whites and native Hawaiians. In that study the authors additionally investigated the association between diet, haplotype and colorectal adenoma and none of these associations reached statistical significance [36].

The two studies conducted on the Korean population, one for gastric and the other for colorectal cancer, differed in their findings. In the first study of gastric cancer, authors also did not observe any association between gastric cancer and AVI/AVI (non-tasters) vs. PAV/PAV (tasters) (OR = 0.995; 95% CI 0.709–1.396, p = 0.272). However, they did observe a significant association of increased risk for developing gastric cancer, which was statistically significant for the comparison of the heterozygous PAV/AVI diplotype relative to PAV/PAV diplotype, (OR = 1.392; 95% CI 1.089–1.780, p = 0.004) [37].

In the second study of colorectal cancer, the reported results for *TAS2R38* and *CA6* were opposite to our current observations, and congruent with our initial hypotheses. In their study, they observed a protective effect of the AVI/AVI diplotype (non-taster) against the PAV/PAV diplotype (taster) (OR = 0.74; 95% CI 0.55–0.98, p = 0.022) with a similar results for PAV/* vs. AVI/AVI (OR = 0.74; 95% CI 0.57–0.96, p = 0.021). Within the same study, the results showed that the combined G/A and G/G genotype (non-tasters) in *CA6* gene reduced the risk of developing colorectal cancer (OR = 0.70; 95% CI 0.55–0.88, p = 0.008). When the two genes (*TAS2R38* and *CA6*) were combined, the strongest protective effect was noted for the AVI/AVIG* genotype (non-tasters) relative to PAV/*A/A (tasters) (OR = 0.50; 95% CI 0.35–0.72. p = 0.001) [38].

In the two previously mentioned Korean studies authors also evaluated if there was an association between having specific bitter taste diplotypes and diet. They did not observe any association between diet and diplotypes in *TAS2R38*, which was explained as being impacted by the addition of condiments to modify the bitter taste of food. This explanation is confirmed by other studies, for example salt is often used to alter the taste of dishes, allowing to neutralize bitter flavor [48]. Fisher at al. using sensory evaluations observed that children who were PROP tasters, would increase their consumption of broccoli by 80% when the vegetables were served with a dip. For children who were bitter taste insensitive no increase in their consumption of vegetables was observed when the vegetables were served with a dip [49]. Also, the method of cooking or preparing dishes can eliminate unpleasant bitter taste of food for individuals with taster haplotype (PAV). Consequently, individuals with a sensitive genotype of *TAS2R38* (PAV*) can eliminate the bitter taste of some vegetables and not be deterred from consuming them, and thus can benefit from these vegetable’s anticarcinogenic properties. By altering the perception of the bitter taste, the impact of a genotype on consumption of bitter tasting vegetables can be minimized, and thus the genotype effect as mediated by consumption of cruciferous vegetables would tend to be null. To our knowledge for *CA6* genotype only one study, in the context of colorectal cancer, evaluated the association between *CA6* A/A genotype and diet with no significant associations observed [38].

Furthermore, the genotype effect of TAS2R38 (PAV*) on various types of cancer can be weak/not detectable due to the fact that other factors may act as mediators between the TAS2R38 (AVI/AVI) and cancer. The impact of alcohol [50, 51] and cigarette smoking [52, 53] on cancer risk is documented in the literature. Also it has also been observed in the literature, though again results are not consistent, that smoking and alcohol consumption, are more common among the AVI haplotypes (non-tasters) [8, 38, 54–60], thus potentially increasing the risk of cancer in such individuals. Therefore, it may be possible that various factors (brassica vegetables and alcohol or/and smoking) counteract each other’s effects on cancer risk, but to draw such conclusions, studies considering the joint effects of these factors and how the bitter tasting/non-tasting genotype, mediates their effect on BC risk, need to be conducted.

Explanation of our results of significant association between rs2274333 and the increased risk of breast cancer for the non-tasters, may be related to other functions of the *CA6* gene than taste perception, such as acid-base balance. The *CA6* gene belongs to the family/group of carbonic anhydrase enzymes containing zinc in the active center. Anhydrases are responsible for maintaining a constant pH in various tissues and catalyze the reversible reaction CO₂ +H₂O↔H^+^+HCO_3_^-^ [61]. Numerous studies have shown that carbonic anhydrases are fundamental to the dynamics of both intracellular and extracellular pH values [62, 63].

Maintaining the correct pH of both intracellular and extracellular pH of the cell is extremely important for cell homeostasis. Therefore, the enzymes responsible for maintaining the pH of the cell seem to be of particular interest from the point of view of cancer development and metastasis. Carbonic anhydrases are such a group of proteins. Two carbonic anhydrases CA9 and CA12 have already been identified as prognostic factors for metastasis and survival [64–66]. However, the function of *CA6* gene in cancer is not known, although it has been observed that the secreted protein of *CA6* gene is downregulated in the human breast cancer tissue [64] and thus can also impact the pH homeostasis. In our study we did not test expression of *CA6* but women with a less active form of the *CA6* gene (G allele) had an increased risk of breast cancer. Perhaps this less active form of the allele of *CA6* jointly with the genetic background of Polish women contributes to the increased risk of breast cancer in this population.

It is worth emphasizing that the genotype of *CA6* affects the structure of the enzyme and its active center. Zinc is a cofactor for CA6, meaning this metal is necessary for the proper and efficient functioning of the active center of this enzyme. Zinc is provided through the diet, and thus its availability in the body may influence the course of enzymatic reactions, so the proper functioning of the enzyme depends on the gene variant and diet.

In summary, the results from studies evaluating the association between the *TAS2R38* and *CA6* genes and the risk of cancer are inconsistent. The lack of replication of genetic associations studies among different populations is a common problem. These discrepancies between the study’s results could be attributable to genetic background, allele frequency, diversity of the population and sample size [67]. It should be emphasized that described studies in our Discussion, involved various cancers and were conducted in various populations, including distant ones, such as the Asian and Caucasian populations, where the frequencies of haplotypes and alleles of these two genes differ.

The frequency of the two main, uncommon and rare haplotypes of the *TAS2R38* gene in the European population is PAV—45.66%; AVI—49.22%; AAV—3.56%; AVV—0.49%; PAI—0.32% PVI—0.03%; AAI—0.55%; PVV—0.17% vs. the Asian population PAV—64.51%; AVI—35.31%; AVV—0.17%. The frequencies for the two alleles of the CA6 gene in the East Asia population, which includes Japan and Korea are A = 0.44%, G = 0.55%, vs. the European population A = 0.70%, G = 0.30%. [7, 68]. In our study, in controls, the distribution of the PAV haplotype and CA6 allele was similar to European population: PAV 41.39%; AVI 55.56%; AAV 2.51%; AVV—0.50%., and in CA6 it was A = 0.71%, G = 0.29%.

Epidemiological studies indicate that about 4–5% of breast cancers are caused by a high penetrance genes, thus the remaining cancer risk, from the perspective of genetically explicable risk may be due to the combination of many low penetrance gene variants [69, 70]. It is well recognized that a combination of individual variants and their interactions with the environment can modulate cancer susceptibility [71]. It should be noted that the alleles and haplotypes in the two studied genes are common variants (MAF>0.05) in both the Asian and European populations, which allows us to conclude that these genes are genes with small effect size and singularly would be associated with a small increase in breast cancer risk. Thus, capturing such association in epidemiological studies requires a large number of participants, and even then, is not always feasible.

However, the complexity of conducting gene-gene, gene-environment interactions on breast cancer risk is discussed in detail by Collins and Politopoules [72] and Travis et al. [73]. Mediation and moderation analyses of environmental factors on disease outcome, as described by Baron and Kenny [40] and Krause et al. [74] indicate that such factors may have opposite effects on outcome of interest, and consequently making the genetic associations with disease outcome as mediated/moderated by the environmental factors, difficult to predict. Research evaluating the effects of the environmental, behavioral and/or physiological characteristics of women’s health status, jointly with genetic information, will increase our knowledge of how this complex interplay between genetics and various risk factors influences breast cancer risk.

## Conclusions

In conclusion, our study did not demonstrate a significant association between the frequency of PAV (tasters) and AVI (non-tasters) haplotypes in the *TAS2R38* and breast cancer risk. For the *CA6* gene, we observed an opposite effect than hypothesized, i.e., the risk of breast cancer was increased for those with the non-taster allele G. Certainly, the association of these genes and cancer risk is influenced by many factors, with diet, and bitterness of specific vegetables influencing their consumption, being one such factor. However, as observed in the literature, the bitterness of the specific vegetables can be mitigated by their preparation. Therefore, their intake may not necessarily be influenced by the genetic predisposition, and thus any given, study might not be able to detect such associations. Future studies which will include a detailed description of dietary patterns of individuals in the context of their genotypes for these two genes will be better equipped to evaluate if there is an association between the variants in these taste genes, individual’s diet and their cancer risk.

## Supporting information

S1 Table Frequency of haplotypes in TAS2R38 gene (with rare haplotypes*). (PDF)

S1 File Raw data. (XLSX)
