# Supplementary material for: Association of the bitter taste genes TAS2R38 and CA6 and breast cancer risk; a case-control study of Polish women in Poland and Polish immigrants in USA
Source: PLoS One. 2024 Apr 30;19(4):e0300061. doi: 10.1371/journal.pone.0300061 (PMC11060581; doi:10.1371/journal.pone.0300061)
Supplement: S1 Table — (PDF) [file pone.0300061.s001.pdf]

S1 Table. Frequency of haplotypes in TAS2R38 gene (with rare haplotypes*)

Polish women in Poland				Polish immigrant women in USA			
Case		Control		Case		Control	
Haplotype	%	Haplotype	%	Haplotype	%	Haplotype	%
AVI	58.2	AVI	57.1	AVI	56.6	AVI	53.1
PAV	36.9	PAV	39	PAV	40.4	PAV	45.2
AAV	4.5	AAV	3.2	AAV	3	AAV	1.4
AVV	0.2	AVV	0.7			AVV	0.3
AAI	0.2						

*Empty cells indicate that a given haplotype was not observed in the population
